# Downregulation of survivin expression and concomitant induction of apoptosis by celecoxib and its non-cyclooxygenase-2-inhibitory analog, dimethyl-celecoxib (DMC), in tumor cells in vitro and in vivo

**DOI:** 10.1186/1476-4598-5-19

**Published:** 2006-05-18

**Authors:** Peter Pyrko, Nathaniel Soriano, Adel Kardosh, Yen-Ting Liu, Jasim Uddin, Nicos A Petasis, Florence M Hofman, Ching-Shih Chen, Thomas C Chen, Axel H Schönthal

**Affiliations:** 1 Department of Pathology, University of Southern California, Los Angeles, USA; 2 Department of Molecular Microbiology and Immunology, University of Southern California, Los Angeles, USA; 3 Department of Chemistry, University of Southern California, Los Angeles, USA; 4 Division of Medical Chemistry and Pharmacognosy, The Ohio State University, Columbus, USA; 5 Department of Neurosurgery, University of Southern California, Los Angeles, USA

## Abstract

**Background:**

2,5-Dimethyl-celecoxib (DMC) is a close structural analog of the selective cyclooxygenase-2 (COX-2) inhibitor celecoxib (Celebrex^®^) that lacks COX-2-inhibitory function. However, despite its inability to block COX-2 activity, DMC is able to potently mimic the anti-tumor effects of celecoxib in vitro and in vivo, indicating that both of these drugs are able to involve targets other than COX-2 to exert their recognized cytotoxic effects. However, the molecular components that are involved in mediating these drugs' apoptosis-stimulatory consequences are incompletely understood.

**Results:**

We present evidence that celecoxib and DMC are able to down-regulate the expression of survivin, an anti-apoptotic protein that is highly expressed in tumor cells and known to confer resistance of such cells to anti-cancer treatments. Suppression of survivin is specific to these two drugs, as other coxibs (valdecoxib, rofecoxib) or traditional NSAIDs (flurbiprofen, indomethacin, sulindac) do not affect survivin expression at similar concentrations. The extent of survivin down-regulation by celecoxib and DMC in different tumor cell lines is somewhat variable, but closely correlates with the degree of drug-induced growth inhibition and apoptosis. When combined with irinotecan, a widely used anticancer drug, celecoxib and DMC greatly enhance the cytotoxic effects of this drug, in keeping with a model that suppression of survivin may be beneficial to sensitize cancer cells to chemotherapy. Remarkably, these effects are not restricted to in vitro conditions, but also take place in tumors from drug-treated animals, where both drugs similarly repress survivin, induce apoptosis, and inhibit tumor growth in vivo.

**Conclusion:**

In consideration of survivin's recognized role as a custodian of tumor cell survival, our results suggest that celecoxib and DMC might exert their cytotoxic anti-tumor effects at least in part via the down-regulation of survivin – in a manner that does not require the inhibition of cyclooxygenase-2. Because inhibition of COX-2 appears to be negligible, it might be worthwhile to further evaluate DMC's potential as a non-coxib alternative to celecoxib for anti-cancer purposes.

## Introduction

Nonsteroidal anti-inflammatory drugs (NSAIDs) have long been implicated in the treatment or prevention of various types of cancer. The biochemical mechanism generally ascribed to this effect is the inhibition of cyclooxygenase (COX) enzymes, which catalyze the initial step in prostaglandin synthesis [1-3]. The traditional NSAIDs, such as flurbiprofen, indomethacin, or sulindac, are able to inhibit both COX-1 and COX-2 enzymes, while new generation drugs, such as celecoxib (Celebrex^®^), valdecoxib (Bextra^®^), or rofecoxib (Vioxx^®^), inhibit only COX-2. Due to their more selective function, these latter drugs, referred to as coxibs, initially had promised to offer the therapeutic benefit of traditional NSAIDs with less of the associated side effects [4-7]; however, this expectation has come under intense scrutiny and has generated considerable controversy in the recent past [8-10].

Celecoxib is widely prescribed under the trade name Celebrex^® ^for relief of symptoms of osteoarthritis and rheumatoid arthritis and was also approved as an adjunct to standard care for patients with familial adenomatous polyposis (FAP). It is suspected that this drug might be useful for the prevention and treatment of colorectal and possibly other types of cancer, and several clinical trials are ongoing to confirm this expectation. In addition, celecoxib has demonstrated potent anti-cancer activity in various animal tumor models in the laboratory [11-17]. Despite these promising results, however, the underlying molecular mechanisms by which celecoxib exerts its anti-tumor potential are not completely understood, in particular because of numerous reports describing potent anti-proliferative and pro-apoptotic effects of this drug in the absence of any apparent involvement of COX-2 [18-24].

In order to investigate the COX-2 independent anti-tumor mechanisms of celecoxib in greater detail, we and others have generated close structural analogs of this compound that lack the ability to inhibit COX-2 activity [25-28]. One such analog is 2,5-dimethyl-celecoxib (DMC), a compound that was first developed in the laboratory of Ching-Shih Chen at Ohio State University [26,28]. Intriguingly, despite its inability to inhibit COX-2, DMC is able to faithfully mimic – without exception – all of celecoxib's numerous anti-tumor effects that have been investigated so far, including the reduction of neovascularization and the inhibition of experimental tumor growth in various in vivo tumor models [21,25,26,28-32]. Therefore, DMC appears to be well suited for studies intended to illuminate the COX-2 independent anti-tumor effects of celecoxib [33].

Because celecoxib and DMC are potent inducers of apoptosis, we investigated their effects on survivin, which is a member of the inhibitor of apoptosis (IAP) family of proteins that has been implicated in the control of cell division and apoptosis [34]. Survivin's function in mitosis is to preserve the mitotic apparatus and to allow normal mitotic progression, whereas its anti-apoptotic function is executed via its ability to prevent caspase activation. The protein is usually not expressed in differentiated normal adult tissues, but is elevated in the majority of human cancers, with very high levels generally being predictive of tumor progression and poor prognosis. In addition, survivin appears to be involved in tumor cell resistance to some anticancer agents and ionizing radiation (for detailed references, see reviews [35-37].

As the above-described characteristics established survivin as a potential target for anticancer therapy, we investigated whether the expression of this anti-apoptotic protein could be restrained by celecoxib and DMC. Here we report that both drugs are able to down-regulate survivin expression and induce apoptosis in numerous tumor cell lines. These effects are not restricted to in vitro conditions, but also take place in drug-treated animals in vivo, where both drugs repress survivin and induce apoptosis in xenograft tumor tissue. Thus, in consideration of survivin's recognized role as a guardian of tumor cell survival, our results suggest that celecoxib and DMC might exert their cytotoxic anti-tumor effects at least in part via the down-regulation of survivin. Because DMC lacks COX-2 inhibitory function, these anti-tumor effects appear to take place without the involvement of celecoxib's well-known target, cyclooxygenase-2.

## Results

### Celecoxib and DMC down-regulate survivin protein levels

**Figure 1:**
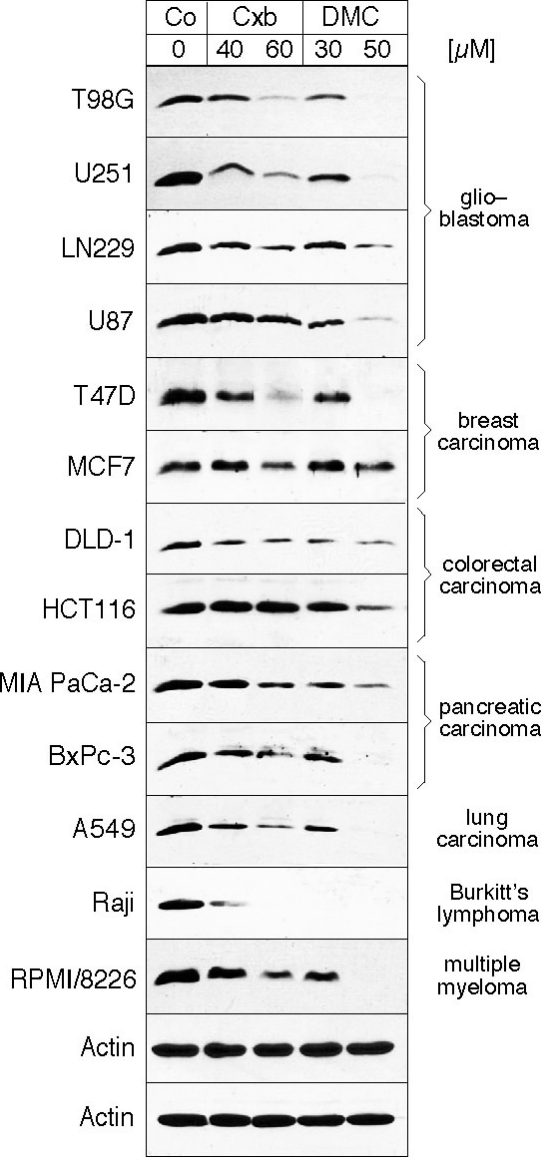
**Celecoxib and DMC decrease levels of survivin protein in various cancer cell lines**. Several different cancer cell lines were cultured in the presence of celecoxib (Cxb) and DMC for 48 hours as indicated. Total cellular lysates were prepared and analyzed by Western blot analysis with specific antibodies to survivin. As a control for equal loading, all blots were also analyzed with antibodies to actin (only two of these control blots are shown at the bottom). The tumor type of each cell line is indicated on the right.

To determine whether celecoxib and DMC would be able to affect survivin expression in a variety of human tumor types, we treated a collection of derived cell lines with either drug in vitro. Because it had been established earlier that DMC is generally more potent than celecoxib, we used 30 and 50 μM of DMC, and 40 and 60 μM of celecoxib. As shown in Figure 1, both drugs were able to down-regulate survivin expression in all cell lines investigated, which included cells derived from glioblastoma, lymphoma, multiple myeloma, and carcinoma of the breast, colon, and prostate. Consistent with earlier studies on other targets, DMC exerted stronger effects than celecoxib and caused a more potent down-regulation of survivin. Although this effect was observed in all cell types, the overall magnitude of down-regulation varied between individual cell lines; for example, whereas Raji lymphoma, T98G glioblastoma, and T47D breast carcinoma cells displayed a very strong down-regulation of survivin, LN229 glioblastoma, MCF7 breast carcinoma, and HCT116 colon carcinoma showed a weaker response at the same concentrations. However, further increased concentrations of these two drugs invariably led to complete downregulation of survivin expression in all cell lines examined, i.e., 60–70 μM DMC or 70–80 μM celecoxib completely suppressed survivin expression, which was accompanied by severe cytotoxicity (not shown).

### Down-regulation of survivin is independent of p53

**Figure 2:**
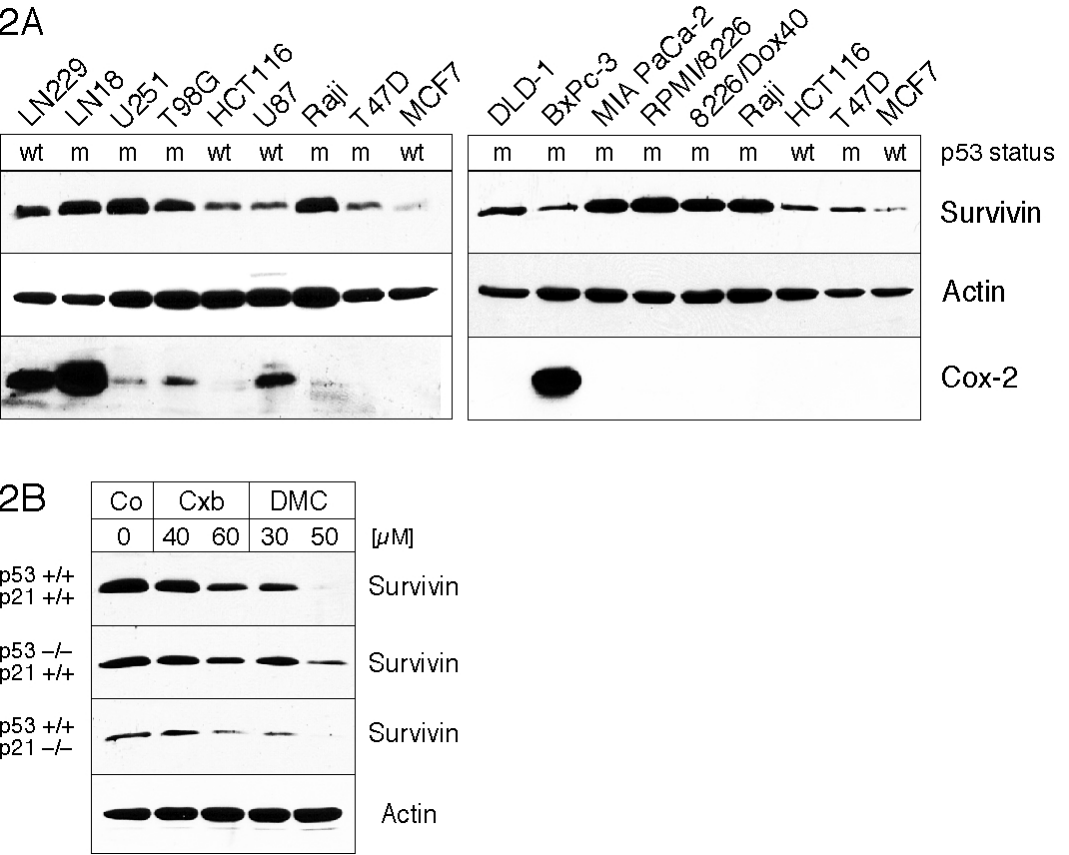
**Basal level expression of survivin and Cox-2 proteins in various cancer cell lines and effect of p53 and p21**. In (A), the various cancer cell lines were cultured in the absence of any drug treatment, harvested in log phase, and analyzed by Western blot analysis with antibodies to survivin, cycloxygenase-2 (Cox-2), and actin (as a loading control). In addition, the p53 status of each line (as reported in a variety of reports) is indicated (wt: wild type; m: mutant). (Note that in LN229 cells, wt p53 function is retained, despite a mutation in the coding sequence.) In (B), three variants of HCT116 colon carcinoma cells were treated with celecoxib (Cxb) or DMC and analyzed by Western blot analysis for survivin levels and actin (as a loading control; only one representative panel is shown). The top panel shows results with HCT116 cells that harbor wild type alleles of the p53 and p21 genes; the second panel is from cells with disrupted p53 alleles (p53-/-); the third panel is from cells lacking p21 (p21-/-).

Because the above results indicated a certain cell type-specific sensitivity with regards to the down-regulation of survivin, we comparatively analyzed several relevant parameters in these cell lines. As it has been shown earlier that the status of the tumor suppressor p53 might influence basal levels of survivin expression [38,39], we investigated whether there was a correlation of p53 status with the basal and/or the differential drug-reduced levels of survivin. As can be seen in Figure 2A, the basal level expression of survivin, i.e., the cellular amount of survivin protein in the absence of drug treatment, varied greatly among the various tumor cell lines. However, overall there was no obvious correlation between this variation of basal level expression and the efficacy of drug-induced repression (compare to Figure 1). But when the mutational status of the p53 gene in these cell lines was investigated from data of the published literature (presented at the top of Figure 2A), and was compared among cell lines of the same tumor type, it appeared that the presence of mutant p53 exerted a small, yet noticeable influence on the efficacy of survivin down-regulation by DMC and celecoxib in some of the cells. For example, in the pair of breast carcinoma cell lines MCF7 (p53 wt) and T47D (p53 mut), T47D displayed a higher basal level (Figure 2) and stronger down-regulation of survivin than MCF7 (Figure 1). The same held true among the various glioblastoma cell lines we investigated: T98G and U251 (both p53 mut) displayed higher basal levels and a somewhat stronger down-regulation of survivin than U87 and LN229 (both p53 wt). Similarly, the colon carcinoma pair HCT116 (p53 wt) and DLD-1 (p53 mut) followed this pattern as well, although in this case the difference was less pronounced.

However, the correlation between p53 status and basal and drug-reduced survivin levels did not hold true in all cell lines. For example, the pair of prostate carcinoma cell lines, MIA-PaCa-2 and Bx-PC-3, displayed a noticeable difference in their basal levels of survivin and in their response to the drugs, even though these cells both harbor mutant p53. Therefore, in order to distinguish whether the observed differential drug responses were indeed related to p53, or rather were an expression of the general genetic heterogeneity of these aneuploid tumor cells, we used an HCT116 colon carcinoma cell line where the p53 gene (or one of its crucial target genes, the cyclin-dependent kinase inhibitor p21^Waf1^, which was found to mediate p53's repression of survivin [40]) was disrupted by targeted homologous recombination [41,42]. As shown in Figure 2B, inactivation of p53 resulted in a minor reduction of drug effects, whereas inactivation of p21 had no effect. Thus, taken together, we conclude that p53 does not play a major role in the observed differential down-regulation of survivin by celecoxib or DMC.

### Down-regulation of survivin is independent of cyclooxygenase-2

Another parameter we decided to analyze in the various tumor cell lines was cyclooxygenase-2 (COX-2). Although the use of DMC, which does not inhibit COX-2, already indicated that this enzyme quite likely played no role in the observed drug effects, we determined the levels of COX-2 protein and investigated whether they would correlate with the sensitivity of these cells to DMC and/or celecoxib. The amount of COX-2 protein was established by Western blot analysis and is shown in Figure 2A. However, when compared to the data presented in Figure 1, we found that cell lines with elevated levels of COX-2 (U87, LN229, Bx-PC-3) did not consistently differ in their extent of survivin down-regulation as compared to cell lines lacking COX-2 (Raji, RPMI/8226, HCT116, MIA-PaCa-2). Thus, as expected, no correlation between COX-2 expression and the degree of survivin down-regulation by DMC or celecoxib was found.

**Figure 3:**
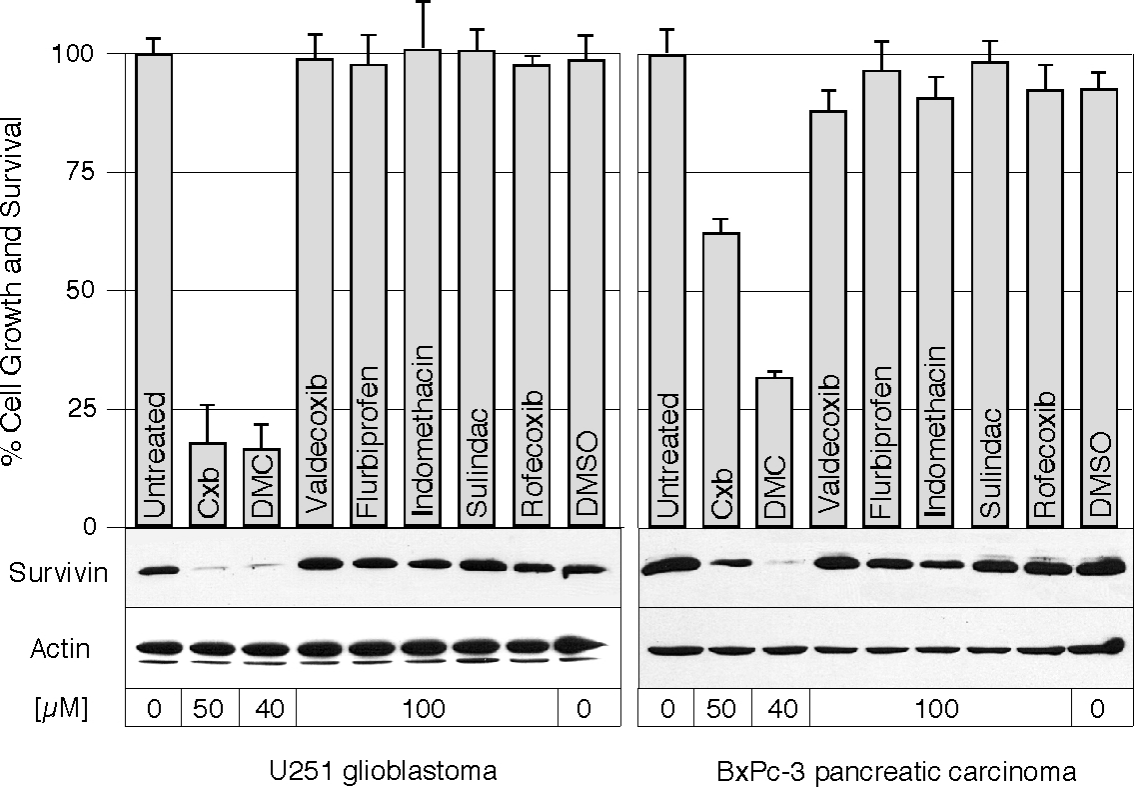
**Downregulation of survivin is specific to celecoxib and DMC and correlates with reduced survival**. U251 glioblastoma or BxPc-3 pancreatic carcinoma cells were cultured in the presence of DMC, various non-steroidal anti-inflammatory drugs (NSAIDs), or solvent DMSO alone, at the concentrations indicated. Cell growth and survival was determined by standard MTT assay (top part of figure). In parallel, total cellular lysates were prepared and analyzed by Western blot analysis with specific antibodies to survivin or to actin as a loading control (bottom part of figure).

The lack of COX-2 involvement was further confirmed by comparing the effects of DMC and celecoxib to other established inhibitors of this enzyme. For instance, flurbiprofen, indomethacin, and sulindac are traditional NSAIDs that inhibit both COX-1 and COX-2, whereas valdecoxib and rofecoxib are coxibs that selectively inhibit only COX-2. When two different tumor cell lines were treated with various concentrations of the above inhibitors, no effect on survivin expression was observed, even at concentrations of up to 100 μM (Figure 3, bottom part), which are more than double the effective concentrations of celecoxib and DMC. Thus, the significant down-regulation of survivin by DMC and celecoxib could not be achieved by comparable concentrations of other COX-2 inhibitors, clearly arguing against an involvement of COX-2 in these processes. In addition, none of these other COX-2 inhibitors was able to substantially impinge on cell growth and survival of these cells (Figure 3, top part), nor were these compounds able to induce apoptosis at these concentrations (not shown). Thus, the differential effects of DMC, celecoxib, and other coxibs and traditional NSAIDs indicated a correlation between the effects on survivin expression and cell survival or death.

### Down-regulation of survivin involves transcriptional repression

**Figure 4:**
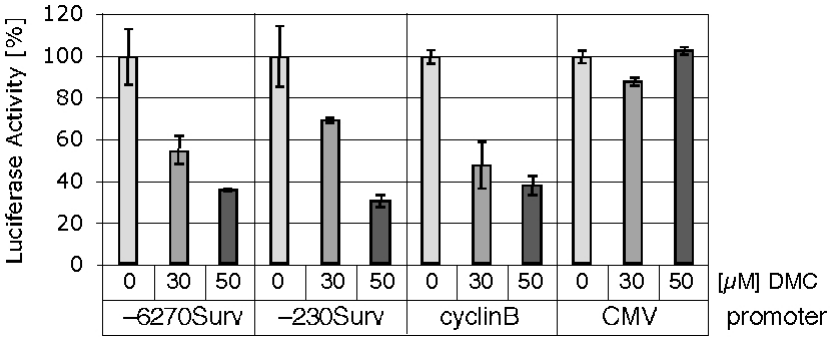
**DMC decreases the activity of the survivin promoter**. Mass cultures of LN229 cells stably transfected with various luciferase reporter constructs under the control of either the survivin promoter (-6270Surv and -230Surv), the cyclin B promoter, or the cytomegalovirus (CMV) promoter, were treated with different concentrations of DMC for 36 hours. Thereafter, cellular lysates were analyzed for luciferase activity. For each reporter construct, basal level activity in the absence of drug at 36 hours was set to 100%. Shown is the mean (± SD; n = 3) luciferase activity from one experiment, which was repeated twice with similar results.

We had shown earlier that celecoxib and DMC are able to inhibit the expression of two key cell cycle-regulatory genes, cyclin A and cyclin B, at the transcriptional level [20,25]. To determine whether survivin expression was similarly affected by these drugs, we generated cells that were stably transfected with luciferase reporter constructs under the control of the survivin promoter. Two different constructs were used; one contained 6270 bp of upstream promoter sequences of the survivin gene, the other only 230 bp. As shown in Figure 4, the activity of both of these constructs was similarly inhibited by DMC and celecoxib (not shown for celecoxib), indicating that these drugs were able to impinge on survivin transcription. As controls, we used a reporter construct under the control of the cyclin B promoter, which, as expected, was down-regulated by DMC as well; however, a luciferase construct under the control of the cytomegaloviral (CMV) promoter was not affected, indicating that DMC (and celecoxib) did not block transcription indiscriminately. Thus, we conclude that, in addition to cyclin A and cyclin B, survivin represents yet another target of these drugs that is affected at the transcriptional level.

### Down-regulation of survivin correlates with increased apoptosis

**Figure 5:**
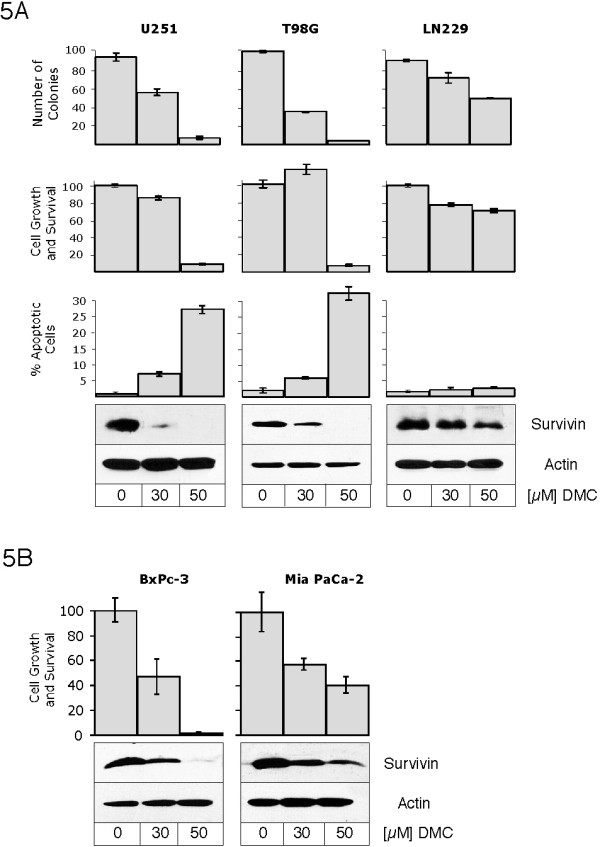
**Downregulation of survivin by celecoxib and DMC correlates with increased apoptosis and reduced cell growth and survival**. The three glioblastoma cell lines U251, T98G, and LN229 (A), or the two pancreatic carcinoma cell lines BxPc-3 and MIA PaCa-2 (B), were treated with 30 or 50 μM DMC or remained untreated for 48 hours. The effects on cell growth/survival and on cell death were determined by various assays. The panels labeled *Number of Colonies *display the results from a colony forming assay, where the number of surviving cells able to spawn a colony of newly grown cells was determined; in this assay, the colonies of adherent cells were stained and visualized with methylene blue two weeks after drug treatment and were counted. The panels labeled *% Cell Growth and Survival *show the results from MTT assays performed at the end of the 48 hour drug treatment period. The panels labeled *% Apoptotic Cells *present the percentage of cells undergoing apoptosis as revealed by the TUNEL assay after 48 hours of drug treatment. At the bottom of each series of panels in A and B, the level of survivin protein at the end of drug treatment is shown, as determined by Western blot analysis with specific antibodies. Western blots for actin are also shown (as a loading control).

Because survivin has a recognized role as an inhibitor of apoptosis, we next investigated whether and how the observed down-regulation of survivin by DMC would relate to the known ability of this drug to induce apoptosis. We used several different representative cell lines (U251, T98G, and LN229 glioblastoma; BxPc-3 and MIA PaCa-2 pancreatic carcinoma) with differing sensitivities to DMC, and comparatively analyzed their response to 30 and 50 μM DMC. As shown in Figure 5, U251, T98G, and BxPc-3 cells responded quite sensitively; these cells displayed a potent down-regulation of survivin, and at the same time strongly increased apoptosis in combination with greatly reduced survival. On the other hand, at these same concentrations of DMC, LN229 and MIA PaCa-2 cells exhibited only a minor down-regulation of survivin, which correlated with marginally increased apoptosis and a much weaker effect on overall cell survival (Figure 5). Thus, the magnitude of survivin down-regulation caused by DMC closely correlated with the extent of apoptosis and with the degree of short-term growth and survival (as determined by MTT assay), as well as long-term survival (as determined by colony forming ability) of these cells.

### Celecoxib and DMC enhance cell killing by CPT-11

**Figure 6:**
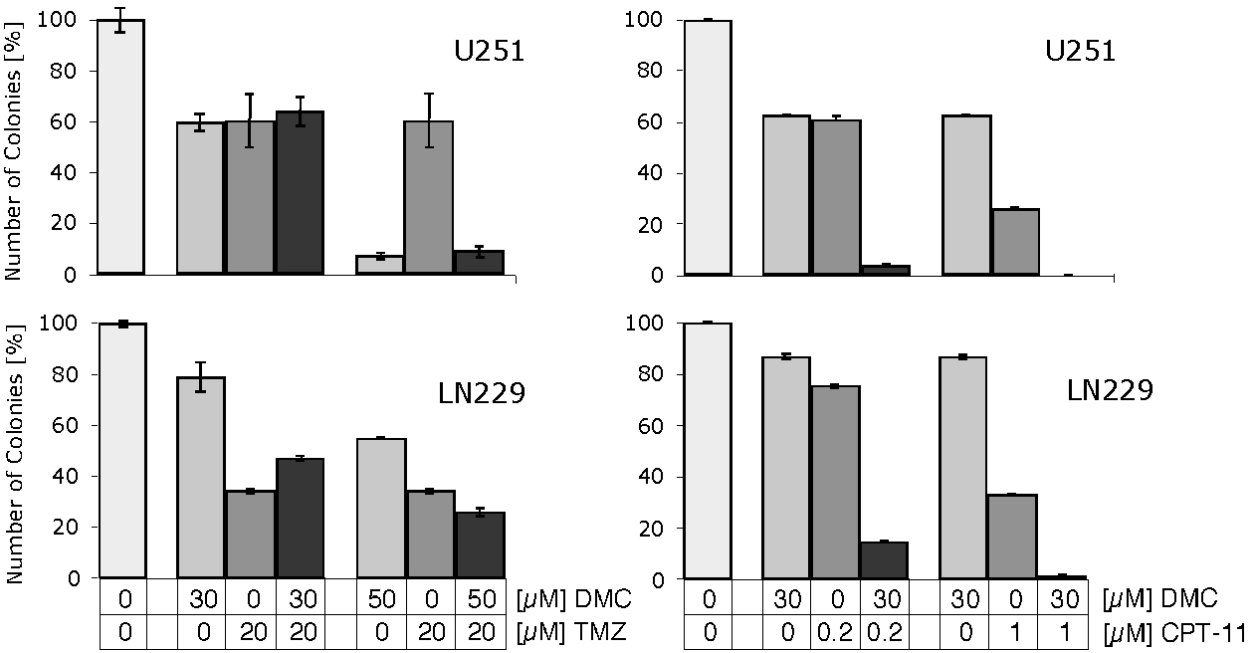
**Combination drug effects of DMC with CPT-11 or temozolomide**. U251 and LN229 glioblastoma cells were treated with DMC, CPT-11, and temozolomide (TMZ) either alone or in combination as indicated for 48 hours. The percentage of surviving cells was established by the conventional colony forming assay, where the number of surviving cells able to spawn a colony of newly grown cells was determined two weeks after drug treatment. Shown are the results from one experiment performed in triplicate, which was repeated several times with very similar results.

With the use of the U251 and LN229 glioblastoma cell lines, we next investigated whether DMC would be able to synergize with other chemotherapeutic drugs to achieve increased tumor cell killing. For this purpose, we used irinotecan (CPT-11) and temozolomide as two representative drugs that are commonly used for the treatment of high-grade brain tumors [43] and determined tumor cell survival with the use of the colony forming assay. Intriguingly, while DMC dramatically increased the cytotoxicity of CPT-11, no such enhancing effect was observed in combination with temozolomide (Figure 6). Furthermore, the outcome was the same in both cell lines, U251 and LN229, which are known to differ in the status of their p53 and PTEN tumor suppressor genes [44,45] (and probably a few other genes as well). Thus, while this result established that DMC is able to cause substantial chemosensitization of glioblastoma cells with different genetic backgrounds, it also revealed that this effect apparently does not take place indiscriminantly with any type of anticancer drug.

### Celecoxib and DMC down-regulate survivin and induce apoptosis in vivo

**Figure 7:**
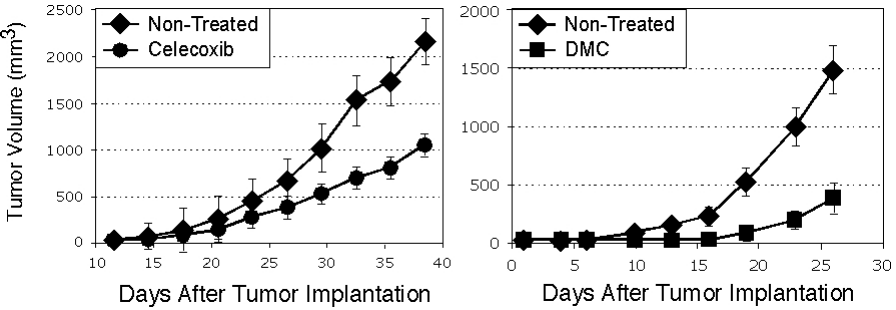
**Inhibition of tumor growth by celecoxib and DMC in vivo**. Nude mice were implanted subcutaneously with U87 glioblastoma cells, and two weeks later received daily chow supplemented with celecoxib, DMC, or no drug. Shown here is the increase in tumor volume over time (mean ± SD; n = 8). At the end of the experiment, the difference in mean tumor volume between the non-treated groups and the groups receiving celecoxib or DMC was statistically significant (p < .01 and p < .003, respectively). Shown are two independent experiments that were performed at different times with different batches of U87 cells and different shipments of animals; therefore, a direct comparison between animals that received celecoxib and animals that received DMC is not possible.

Finally, we investigated whether the effects of DMC and celecoxib on survivin expression would also take place in vivo. For this purpose, we used a xenograft nude mouse tumor model with subcutaneously implanted glioblastoma cells. After palpable tumors had developed, the animals received chow supplemented with either celecoxib, DMC, or no drug (control group). As shown in Figure 7, the group of animals that were treated with either celecoxib or DMC displayed significantly (p < .01 and p < .003, respectively) reduced tumor growth as compared to the group of untreated animals, which was in keeping with similar results published with the use of prostate carcinoma and Burkitt's lymphoma xenograft mouse tumor models [21,25].

**Figure 8:**
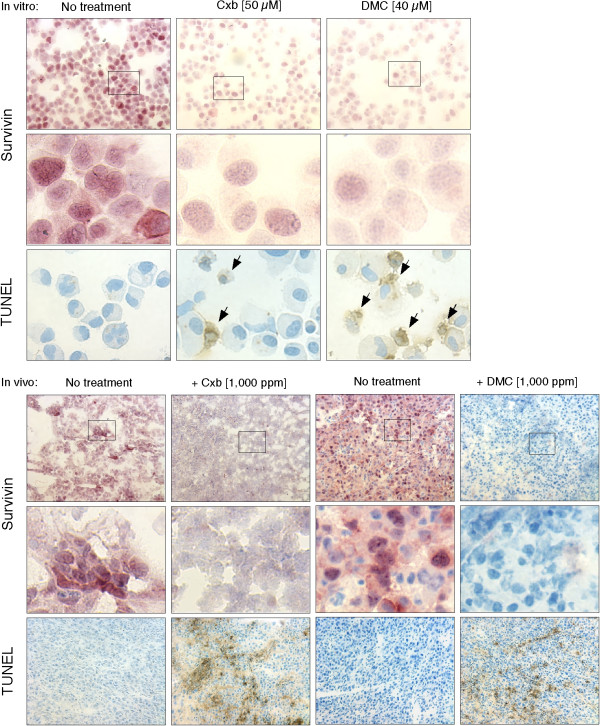
**Downregulation of survivin by celecoxib and DMC correlates with increased apoptosis in vitro and in vivo**. Top half: U87 glioblastoma cells were treated with celecoxib (Cxb) or DMC for 48 hours in vitro; thereafter, cytospins were performed and the cells were subjected to immunohistochemical analysis of survivin protein levels and, in parallel, TUNEL assay for apoptotic cell death. Bottom half: tumor sections from animals described in Figure 7 were analyzed by immunohistochemistry for survivin expression and by TUNEL assay for apoptotic cell death. In all cases, representative sections are shown. Small black rectangles denote enlarged areas of the same photograph shown below. Arrows indicate examples of TUNEL-positive, i.e., apoptotic, cells.

Representative tumors were collected from the animals and analyzed by immunohistochemistry for survivin expression and with the TUNEL assay for the presence of apoptotic cell death. Typical results from the staining of numerous tumor sections are presented in Figure 8 (bottom half). For comparative purposes, we also performed the same type of analysis on glioblastoma cells cultured and treated with drugs in vitro (see top half of Figure 8). Under in vitro conditions, and in keeping with the results shown further above, celecoxib and DMC caused substantial reduction of survivin expression, and at the same time, increased levels of apoptotic cell death (Figure 8, top). Tumor tissue obtained from control (non-drug treated) animals stained strongly positive for survivin protein, and at the same time, was apparently negative for the presence of apoptotic cell death (Figure 8, bottom). In contrast, tumor tissue from drug-treated animals displayed drastically reduced levels of survivin, to the point where not a single positive cell could be found in tumors from DMC-treated animals. Concomitantly, the tumor tissue from drug-treated animals stained clearly positive for the presence of apoptotic cell death (Figure 8, bottom). Thus, in agreement with the findings obtained in vitro, we found that in vivo as well, both drugs were able to suppress survivin expression and concomitantly induce apoptosis in tumor tissue.

## Discussion

The selective COX-2 inhibitor celecoxib appears to hold promise for the treatment and prevention of colorectal cancer and possibly for other cancers as well. Because COX-2 is an oncogene [46] and over-expressed in a large number of tumors, it is generally thought that the COX-2-inhibitory function of celecoxib is critical for its anti-tumor property [4,47-49]. However, several recent studies [19,21-24,27,50], including from our laboratory [20,51], have indicated that celecoxib might be unique among the class of coxibs because this particular compound appears to be able to also suppress tumor formation in the absence of COX-2 involvement. For example, all coxibs completely inhibit COX-2 at very low micromolar concentrations in cell culture; yet only celecoxib causes efficient growth arrest and induction of apoptosis at low concentrations – an effect that is furthermore independent of the amount, or even the presence, of intracellular COX-2 (i.e., it takes place even in cells that lack COX-2 protein) [20,23,26,30,50,52-54]. Additional strong support for COX-2-independent anti-tumor effects of celecoxib has come from the use of its close structural analog, 2,5-dimethyl-celecoxib (DMC) (.)[33], which lacks COX-2 inhibitory function, yet was shown to faithfully mimic the anti-tumor effects of celecoxib in various experimental systems, including the reduction of neovascularization and the inhibition of experimental tumor growth in prostate carcinoma and Burkitt's lymphoma xenograft mouse tumor models [21,25,26,28-32].

The underlying mechanisms of celecoxib's (and DMC's) COX-2 independent anti-tumor effects are not completely understood, although several non-COX-2 targets have been described that are affected by these two drugs in vitro and in vivo [21,25-28,31,32]. In the present report, we demonstrate that survivin, a protein that is critically involved in the regulation of mitosis and the protection of cells from apoptosis, is potently down-regulated by celecoxib and by DMC in all tumor cell lines examined. This effect appears to be independent of any involvement of COX-2, as indicated by three observations: (i) both drugs down-regulate survivin even in cells that do not express detectable amounts of COX-2 (Figure 2A); (ii) none of the other COX inhibitors tested, including the coxibs rofecoxib (Vioxx) and valdecoxib (Bextra), are able to impinge on survivin expression (Figure 3); (iii) DMC does not inhibit COX-2, yet potently down-regulates survivin as well.

There are a few reports from other groups [55-58] indicating that, in addition to celecoxib, some other NSAIDs appear to be able to reduce survivin expression, and these findings could be viewed as being discrepant to ours. However, much higher concentrations were required; for example, Zhang et al. [58] applied 200 μM of sulindac, and Lin et al. [57] used 300 μM of etodolac to impact survivin expression. Compared to our results presented here, these reports further emphasize our observation that celecoxib and DMC are unique in that these two drugs are able to suppress survivin expression at significantly lower concentrations than other NSAIDs. Furthermore, studies with the use of non-small cell lung cancer (NSCLC) cell lines have indicated that increased COX-2 activity might contribute to the stabilization of survivin in these cells [59,60]. While these reports indicate a role of COX-2 in the expression of survivin, it appears that this observation cannot be generalized, as we have not observed a correlation between COX-2 activity and the expression levels of survivin in the various tumor cells lines used in our study (Figure 2).

The potent down-regulation of survivin by celecoxib and DMC, but not by other COX inhibitors, is reminiscent of earlier reports demonstrating that only celecoxib and DMC, but not other COX inhibitors, are able to efficiently induce apoptosis at comparatively low concentrations [21,25,26,28]. This correlation suggests that survivin might be an important mediator of the cell death-inducing function of celecoxib and DMC. Indeed, when we compared the kinetics of survivin down-regulation with the resulting increase in apoptosis in two cell lines with varying sensitivities to DMC (Figure 5), we noticed a very close correlation between the degree of survivin down-regulation and the induction of apoptosis. In these cases, stronger down-regulation of survivin by DMC was associated with substantially more efficient induction of apoptosis. These results are also consistent with our observation (Figure 3) that those NSAIDs that did not affect survivin expression (rofecoxib, valdecoxib, flurbiprofen, and others) also did not impinge on cell growth and survival and did not induce apoptosis.

In addition to survivin, there are several other intracellular proteins that are known to restrain cell death when highly expressed, such as, for example, Bcl-2, Bcl-xL, c-IAP2, XIAP, and FLIP, which also have been found overexpressed in some tumors [61]. While our study did not investigate the potential contribution of these components, studies by others have excluded the involvement of Bcl-2, Bcl-xL, Bax, Bad, or Bak in the apoptosis-stimulating mechanisms of celecoxib and several of its derivatives, and instead provided evidence that these drugs appear to function via the disruption of the mitochondrial membrane potential [62]. This latter observation is of particular relevance, as it has been demonstrated that suppression of survivin expression by RNA interference causes loss of mitochondrial membrane potential and spontaneous apoptosis [63]. Taken together, these data consistently support our view that the observed down-regulation of survivin by celecoxib and DMC might constitute an important step in the induction of apoptotic cell death by these drugs.

Considering the well-known function of survivin as an inhibitor of caspases and, consequently, as an anti-apoptotic protein [35,64], it is not surprising that down-regulation of this protein by celecoxib and DMC is associated with increased cell death. It has been shown in several other experimental systems that the down-regulation of survivin expression, for example by antisense or siRNA approaches [65], results in elevated "basal level" apoptosis and, perhaps more importantly, causes substantially increased sensitivity of such tumor cells to killing by chemotherapeutic drugs or ionizing radiation (for examples, see [66-71]). From these earlier results, one might expect that the down-regulation of survivin by celecoxib or DMC should sensitize these cells to other cancer drugs. We tested this assumption with two widely used anticancer drugs, CPT-11 (irinotecan; Camptosar^®^) and temozolomide (Temodar^®^). Intriguingly, while DMC vastly increased cell killing by CPT-11, no such enhancing effect was observed after co-treatment with temozolomide. Thus, while these results establish proof-of-principle that DMC can substantially enhance tumor cell killing by other anticancer drugs, this obervation cannot be generalized and certainly deserves further study. In this context, it should be noted that celecoxib has been shown previously to enhance the anti-tumor efficacy of CPT-11 in a xenograft mouse model in vivo [16], and a Phase II study revealed encouraging activity of this drug combination among heavily pretreated patients with recurrent malignant glioma [72]. Considering the apparent mimicry of celecoxib's anti-tumor effects by DMC, it might be worthwhile to explore the combination effects of CPT-11 and DMC in greater detail. The potential advantages of evaluating the non-coxib DMC for use in the clinic will be discussed further below.

Our efforts to understand the mechanisms by which DMC accomplishes the down-regulation of survivin revealed that at least part of this regulation occurs at the level of transcription, i.e., our results clearly indicate that DMC is able to potently inhibit survivin expression at the gene level via the inhibition of promoter activity (Figure 4). The extent of survivin promoter inhibition is comparable to the transcriptional repression of the cyclin A and cyclin B promoters by DMC and celecoxib, which we described earlier and which represents a crucial component of the cell cycle-inhibitory function of these two drugs [20,25]. Thus, similar to the negative regulation of cell cycle components by these two drugs, transcriptional events also appear to be involved in mediating their apoptosis-inducing function (not shown for celecoxib).

Although the above described transcriptional events are quite prominent, additional levels of survivin regulation by celecoxib and DMC are likely. For example, it has been shown that survivin protein is stabilized and protected from degradation via its phosphorylation by the critical cell cycle regulator, cyclin-dependent kinase (CDK). In particular, phosphorylation on threonine-34 of the survivin protein, which is accomplished by the cyclinB/cdk1 complex, leads to substantial extension of survivin's half-life during mitosis [73,74]. Conversely, it has been shown that the inhibition of cyclinB/cdk1 activity by various modes of intervention leads to increased turn-over and loss of survivin protein [75-78]. In this regard, we have recently demonstrated that the transcriptional down-regulation of cyclin A and cyclin B by celecoxib or DMC, as mentioned further above, effects the complete loss of enzymatic activity of the respective CDK complexes, including cyclinB/cdk1 [20,25]. Thus, we surmise that in addition to the transcriptional down-regulation of survivin expression, DMC and celecoxib also cause its increased posttranslational degradation via the elimination of CDK enzymatic activity.

In the past, studies investigating the COX-2 independent effects of celecoxib in vitro have been received with reservations, due to the relatively high concentrations of drugs that were required to generate such effects. While drug concentrations between 10 to 80 μM are generally needed to produce anti-proliferative and apoptosis-inducing effects in cell culture in vitro, celecoxib concentrations measured in the serum of patients or animals are in the range of 3–10 μM [79-81]. Thus, this discrepancy has led to the suggestion [17,82] that in vitro effects of celecoxib (and perhaps DMC) might be an artifact and not reflective of the mechanisms taking place in vivo. It was therefore imperative for us to demonstrate whether or not the down-regulation of survivin by celecoxib and DMC could be recapitulated in an in vivo model. As convincingly demonstrated by our results, both celecoxib and DMC were able to potently inhibit survivin expression in tumors of a xenograft mouse tumor model (Figure 8). Even more so, similar to the events in our in vitro system, the number of apoptotic cells in tumors from drug-treated animals was substantially elevated. We therefore believe that those drug-induced events that we documented under elevated drug concentrations in vitro do not represent artifacts of the cell culture system, but rather are reflective of events that also take place in vivo in drug-treated animals.

The experimental use of DMC alongside celecoxib encompasses an important aspect that relates to the recently revealed potentially life-threatening side effects of coxib use in the clinic. The long-term use of coxibs at high dosages – as believed to be necessary if used in anti-cancer therapy – is troubled by severe, potentially life-threatening risks, such as cardiovascular events, renal injury, and gastrointestinal toxicity [9,83-86]. Considering that these side effects are believed to be a class effect due to the inhibition of COX-2 and the resulting imbalance of prostanoids [8,87,88], it is tempting to speculate that the clinical use of a celecoxib analog such as DMC, which lacks COX-2 inhibitory function but maintains anti-tumor potency, perhaps might avoid many of these unwanted side effects – and possibly could be used at even higher dosages than celecoxib for certain anti-tumor purposes.

## Conclusion

It has become clear that at least parts of celecoxib's documented anti-tumor effects are mediated via mechanisms that do not appear to involve COX-2. In this regard, our study presents the anti-apoptotic and chemoprotective protein survivin as an apparently important component that is involved in mediating the drug's COX-2-independent induction of apoptotic tumor cell death. This provides additional evidence that DMC, which does not inhibit COX-2, is able to potently mimic all known anti-tumor functions of celecoxib, and further supports our proposition [33] that it might be worthwhile to further evaluate DMC's potential anti-cancer benefit in the clinic.

## Materials and methods

### Materials

Celecoxib is 4- [5-(4-methylphenyl)-3-(trifluoromethyl)-1*H*-pyrazol-1-yl]benzenesulfonamide [89]. DMC is a close structural analog, where the 5-aryl moiety has been altered by replacing 4-methylphenyl with 2,5-dimethylphenyl, resulting in 4- [5-(2,5-dimethylphenyl)-3-(trifluoromethyl)-1*H*-pyrazol-1-yl]benzenesulfonamide [21,51]. Both compounds were synthesized in our laboratory according to previously published procedures; see ref. [89] for celecoxib and ref. [51] for DMC. Each drug was dissolved in DMSO at 100 mM (stock solution). In the case of valdecoxib [90] and rofecoxib [91], commercial caplets of Bextra^® ^(Pfizer, New York, NY) and Vioxx^® ^(Merck, Whitehouse Station, NJ), respectively, were suspended in H_2_O to disintegrate the excipient, and the active ingredient was dissolved in DMSO at 25 mM. In addition, we used pure rofecoxib powder that was synthesized in our laboratory according to established procedures [92]. All traditional NSAIDs were purchased from Sigma (St. Louis, MO) in powdered form and dissolved in DMSO at 100 mM. All drugs were added to the cell culture medium in a manner that kept the final concentration of solvent (DMSO) below 0.5%.

### Cell lines and culture conditions

Most cell lines were obtained from the American Tissue Culture Collection (ATCC) and were propagated in DMEM or RPMI (GIBCO BRL, Grand Island, NY) supplemented with 10% fetal bovine serum, 100 U/ml penicillin, and 0.1 mg/ml streptomycin in a humidified incubator at 37°C and a 5% CO_2 _atmosphere. The HCT116 colon carcinoma cell line, and derivatives thereof where the p53 tumor suppressor gene or the p21^Waf1 ^gene were disrupted by targeted homologous recombination [41,42], were kindly supplied by Bert Vogelstein, Johns Hopkins Oncology Center (Baltimore, MD). Some of the glioblastoma cell lines were provided by Frank B. Furnari and Webster K. Cavenee (Ludwig Institute of Cancer Research, La Jolla, CA).

### Immunoblots and antibodies

Total cell lysates were prepared by lysis of cells with RIPA buffer [93], and protein concentrations were determined using the bicinchoninic acid (BCA) protein assay reagent (Pierce, Rockford, IL). For Western blot analysis, 50 μg of each sample was processed as described [94]. The primary antibodies were purchased from Cell Signaling Technologies (Beverly, MA), Cayman Chemical (Ann Arbor, MI), or from Santa Cruz Biotechnology, Inc. (Santa Cruz, CA) and were used according to manufacturer's recommendations. The secondary antibodies were coupled to horseradish peroxidase, and were detected by chemiluminescence using the SuperSignal West substrate from Pierce. All immunoblots were repeated at least once to confirm the results.

### Immunohistochemistry

Immunohistochemical analysis of protein expression in tumor tissues and cell lines was performed with the use of the Vectastatin ABC kit (Vector Laboratories, Burlingame, CA) according to manufacturer's instructions. This procedure employs biotinylated secondary antibodies and a preformed avidin: biotinylated enzyme complex that has been termed the ABC technique. As the primary antibody, we used anti-survivin antibody (Santa Cruz Biotech) diluted 1:100 in 2% normal goat blocking serum.

### TUNEL staining

Apoptosis was measured quantitatively with the use of the terminal deoxynucleotidyl transferase (TdT)-mediated dUTP nick end-labeling (TUNEL) assay [95]. All components for this procedure were from the ApopTag In Situ Apoptosis Detection kit (Chemicon, Temecula, CA), which was used according to the manufacturer's instructions.

### MTT assay

MTT assays were performed in 96-well plates as described in detail elsewhere [31] with the use of 3.0–8.0 × 10^3 ^cells per well.

### Plasmids and stable transfections

The human LN229 glioblastoma cell line was stably co-transfected with individual luciferase reporter plasmids and the pSV2neo plasmid. The latter expresses the bacterial aminoglycoside-3'-phosphotransferase (neo) gene [96], which enables selection of transfected cells in medium containing the aminoglycoside G418 sulfate. Stable transfections were performed with the use of Lipofectamine 2000 (Invitrogen, Carlsbad, CA), and mass cultures of transfected cells were selected in G418 according to standard protocols [97].

The following luciferase reporter plasmids were used. Cyclin B-luc harbors 555 base pairs (bp) of upstream cyclin B promoter sequences [98] and was kindly provided by William R. Taylor, Cleveland Clinic Foundation (Cleveland, OH). CMV-luc is under the control of 880 bp encompassing the promoter of cytomegalovirus (CMV) [20]. The survivin reporter plasmids -6270Surv-luc and -230Surv-luc harbor 6270 bp and 230 bp, respectively, of the upstream promoter region of the survivin gene [99] and were kindly provided by the laboratory of Dario Altieri, Yale University (New Haven, CT).

### Tumor growth in nude mice

All animal protocols were approved by the Institutional Animal Care and Use Committee (IACUC) of the University of Southern California, and all applicable policies were strictly observed during the course of this study. Four- to six-week-old male athymic nu/nu mice were obtained from Harlan (Indianapolis, IN) and kept in a pathogen-free environment. To support more consistent tumor take and uniform growth [100, 101], the animals were whole-body irradiated with 300 cGy of ionizing radiation (Cesium 137) four days prior to xenotransplantation by using a low dose-rate laboratory irradiator (Gammacell 40; Atomic Energy of Canada Limited, Canada).

For tumor inoculation, 5 × 10^5 ^U87 glioblastoma cells were injected subcutaneously into the right flank. Once palpable tumors had developed, the animals were randomly divided into three groups: (i) treatment with celecoxib (1,000 ppm in animal chow), (ii) treatment with DMC (1,000 ppm in animal chow), and (iii) no drug treatment (regular chow without drug added). The tumor size in all animals was measured every three to four days. Tumor size was calculated by the following formula: Volume (mm^3^) = L û W û H û 0.5 (L: length, W: width, H: height). Student t-test was used for statistical analysis, and a P-value of <0.05 was considered significant.

## Authors' contributions

PP performed experiments and assembled the manuscript. NS, AK, and Y-TL performed experiments. JU and NAP were responsible for synthesizing the various drugs. C-SC supplied additional samples of drugs and provided guidance for the project. FMH and TCC participated in the design and execution of the project. AHS conceived of the study and participated in its design, execution, and coordination. All authors read and approved of the final manuscript.
